# Fatal exulceratio simplex (dieulafoy lesion) – a case report and review

**DOI:** 10.1007/s12024-024-00895-4

**Published:** 2024-09-19

**Authors:** Luzern Tan, John D. Gilbert, Roger W. Byard

**Affiliations:** 1 Adelaide Medical School, The University of Adelaide, Frome Road, Level 2, Room N237, Helen Mayo North, Adelaide, SA 5005 Australia; 2 Forensic Science South Australia, 21 Divett Place, Adelaide, South Australia 5005 Australia

**Keywords:** Dieulafoy lesion, Exulceratio simplex, Hemorrhage, Death, Stomach, Case report and review

## Abstract

A 64-year-old man involved in a low-speed vehicle crash was found at autopsy to have altered blood extending from his stomach to his rectum. Within the stomach a small arterial vessel opened onto the mucosa of the posterior wall of the antrum adjacent to the pylorus with no adjacent mucosal ulceration or malignancy. Histologic sections showed the typical appearances of a Dieulafoy lesion with a tortuous small arteriole within the submucosa extending to the gastric lumen with an overlying cap of recently formed clot. There were no injuries attributable to the vehicle collision. Death was due to a bleeding Dieulafoy lesion of the stomach with a background of cardiomegaly. Dieulafoy lesion of the stomach is a rare disorder accounting for only 1–2% of cases of acute gastrointestinal hemorrhage. Although its pathogenesis is poorly understood it is capable of producing life-threatening bleeding, as in the present case. The small size of the lesion may make it difficult to identify at the time of autopsy.

## Introduction

Exulceratio simplex, more commonly known as Dieulafoy lesion, is characterised by superficial tortuous arterial vessels within the submucosa of the gastrointestinal tract [1]. Although most commonly occurring in the stomach (especially at the lesser curvature) it can be found in other locations along the gastrointestinal tract, its associated organs and at surgical anastomosis sites [2–4]. Very rarely it may occur at other sites such as the respiratory tract [1, 5]. Although lesions may be congenital in origin [2] or result from mucosal atrophy, the actual pathogenesis remains unclear [3, 6]. While lethal upper gastrointestinal hemorrhage is usually the result of peptic ulceration, esophageal varices or gastritis, uncommon entities such as Dieulafoy lesion need to be considered. The following report of this rare entity demonstrates characteristic features and analyses issues at autopsy that may occur in diagnosis.

## Case Report

A 64-year-old man was found unresponsive slumped in the driver’s seat of his vehicle with airbags deployed. Resuscitation at the scene and at the local hospital was unsuccessful. His past history included hypertension, poorly controlled type II diabetes mellitus, hypercholesterolemia, hypothyroidism, osteoarthritis, obstructive sleep apnea and depression.

**Fig. 1:**
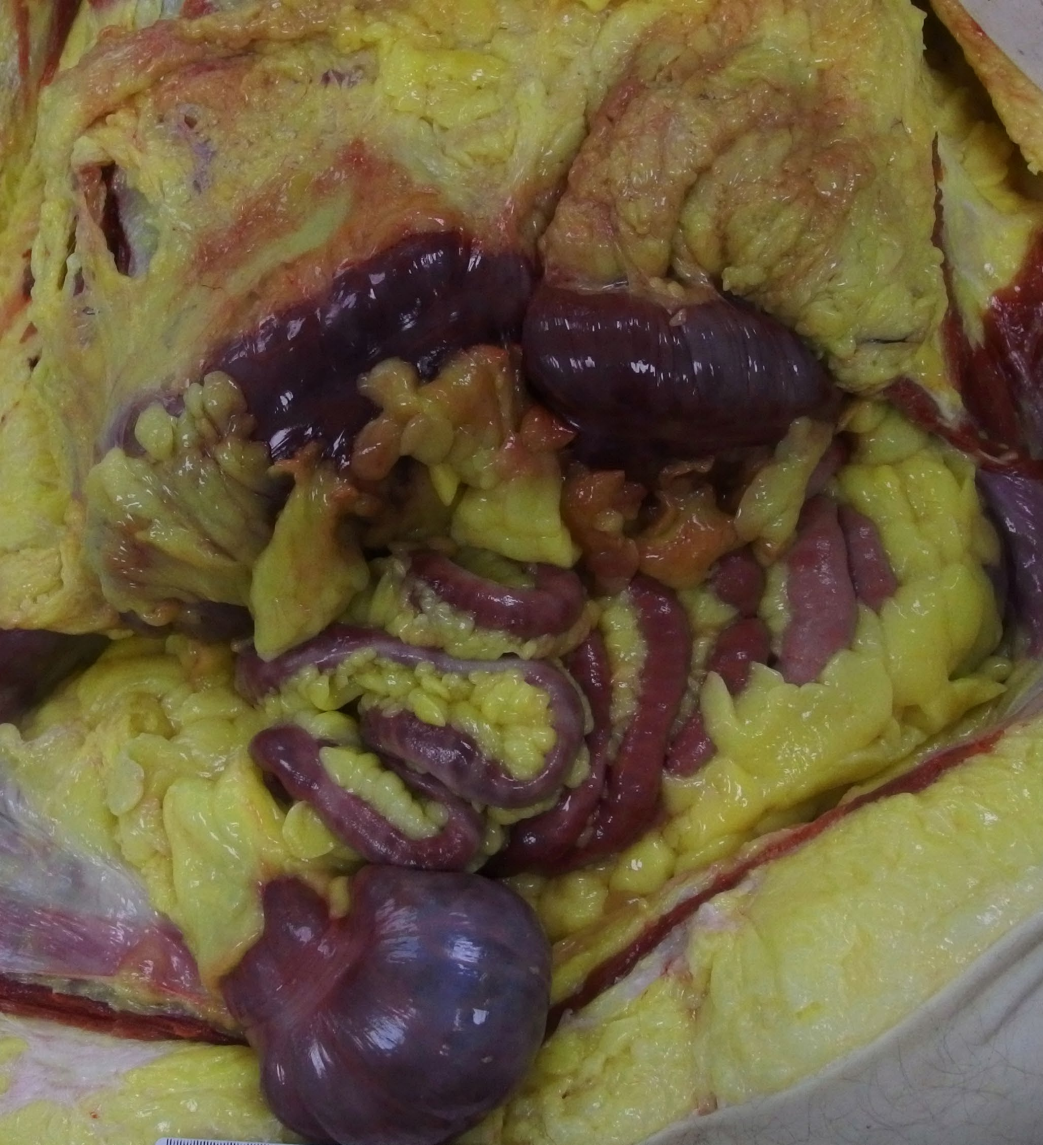
Dark discoloration of the small and large intestines due to filling with altered blood and melena from a bleeding Dieulafoy’s lesion of the stomach

**Fig. 2:**
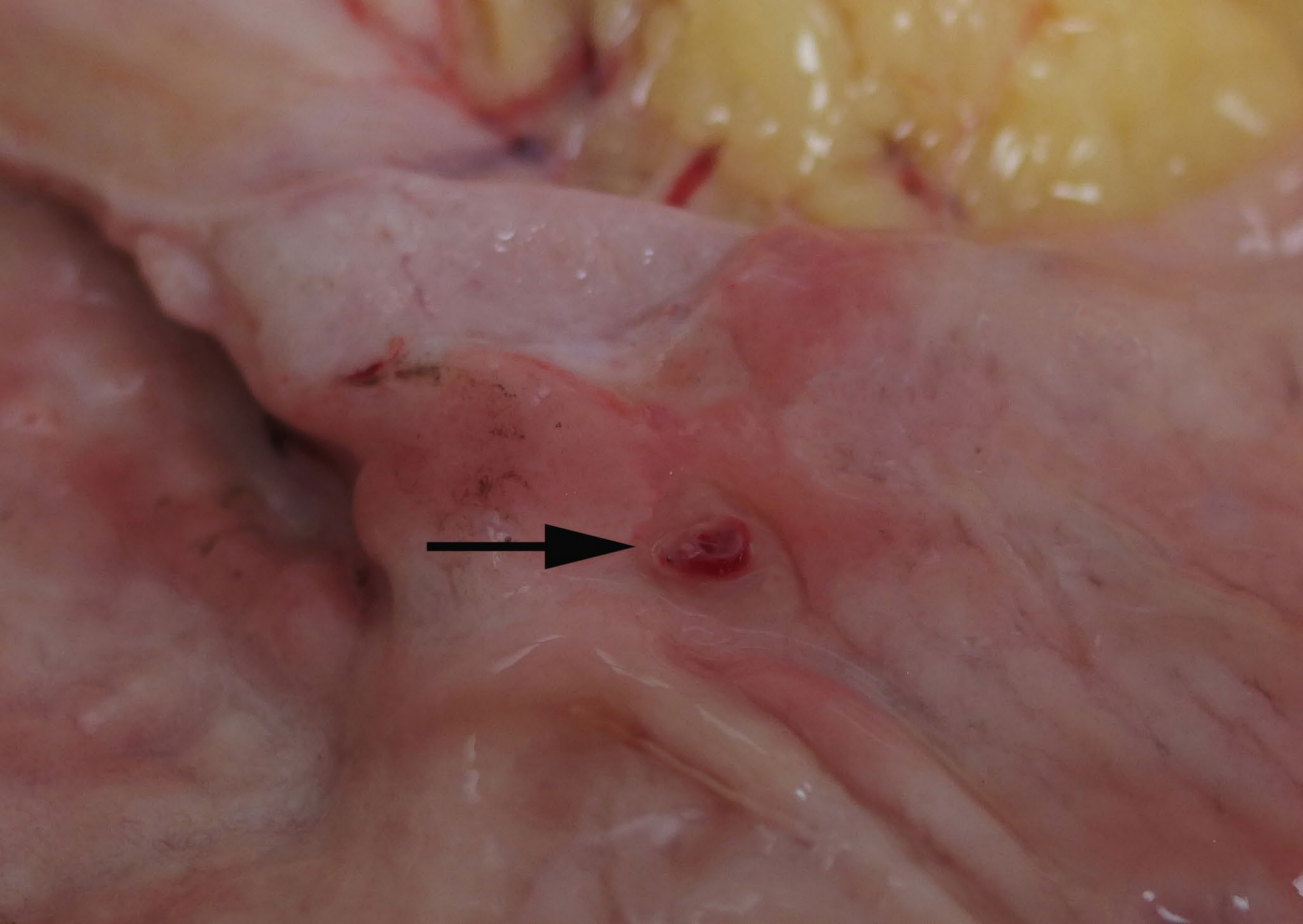
A small vessel opening onto the mucosa of the posterior wall of the antrum adjacent to the pylorus characteristic of Dieulafoy’s lesion (arrow)

**Fig. 3:**
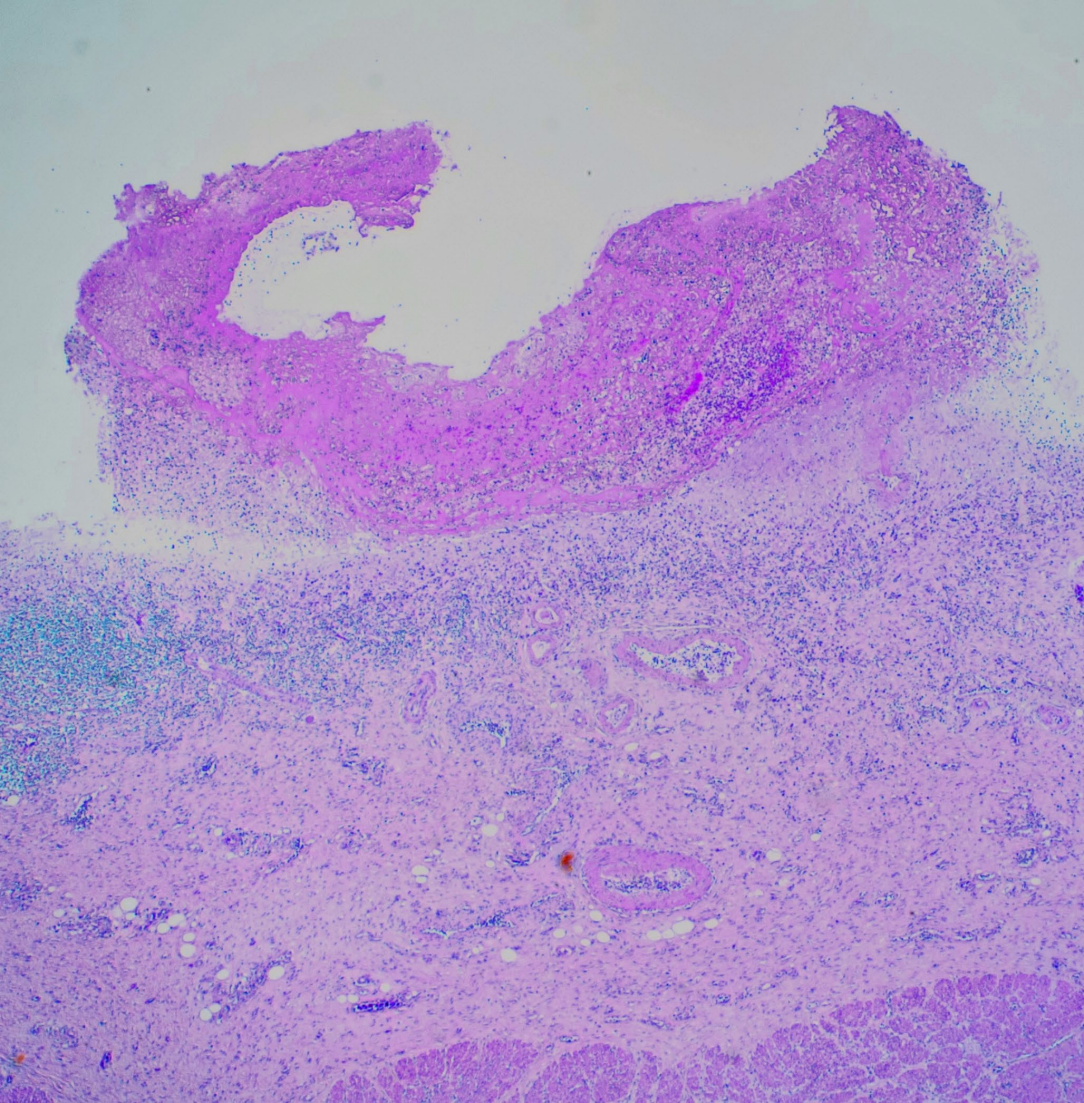
Microscopy of the area of antrum showing a large arterial vessel within the submucosa leading up to the mucosa. An acute inflammatory infiltrate is present with an adherent blood clot. (Hematoxylin and Eosin x 120)

At autopsy the major findings were within the gastrointestinal tract where altered blood was present within the stomach, duodenum and small intestine. The large intestine was also filled with melena that extended from the cecum to the rectum (Fig. 1). The mucosal surfaces of the gut were normal throughout except for the stomach where a small vessel was found opening onto the mucosa of the posterior wall of the antrum adjacent to the pylorus (Fig. 2). There was no macroscopic evidence of mucosal ulceration or malignancy. Histologic sections showed a tortuous small arteriole in the submucosa extending to the gastric lumen with an overlying cap of recently formed clot. The mucosa for several millimetres next to the clot showed loss of normal mucosal glands with acute inflammation. Mild submucosal fibrosis was present around the submucosal vessel (Fig. 3).

Other findings at autopsy included fusion of the left and right coronary cusps of the aortic valve with moderate dystrophic calcification and stenosis. There was no endocarditis. This was associated with concentric left ventricular hypertrophy with a left ventricular free wall thickness of 22 to 25 mm and a heart weight of 633 gms (*N* = 299–521 gms).

There were no injuries attributable to the vehicle collision and no other underlying natural diseases that could have caused or contributed to death. Toxicological analyses of blood revealed no alcohol, with a therapeutic level of citalopram. Death was due to significant gastrointestinal blood loss from a bleeding Dieulafoy lesion of the stomach with a background of cardiomegaly; the latter associated with hypertension and aortic valve stenosis.

## Discussion

Dieulafoy lesions represent an uncommon cause of gastrointestinal bleeding, accounting for only 1–2% of all gastrointestinal cases [3, 4], and approximately 6.5% of those from the upper gastrointestinal tract [4]. They may occur at all ages although are most common in older males (M: F = 2:1) [1, 7, 8]. In pediatric populations there is no male predominance [9]. Although normally asymptomatic they may cause hematemesis, hematochezia or melena, with clinical manifestations ranging from anaemia-induced fatigue to hemovolemia or hemorrhagic shock [3].

The underlying etiology of these lesions is still not well understood with suggestions that the pulsation of an unusually large superficial artery (or arteriole) may disrupt the overlying mucosa and eventually expose the vessel to mechanical and chemical injury [3, 6]. Alternatively, it has been hypothesized that ageing or cardiovascular disease may expose underlying pathology [2, 3], although the affected vessels do not typically have any aneurysmal, arteritic or atherosclerotic changes [1, 3]. Non-steroidal anti-inflammatory drugs (NSAIDs), such as aspirin, and alcohol may be associated with an increased presentation of these lesions due to gastritis and mucosal thinning [1, 3, 4, 7, 8].

Review of the English literature revealed 42 cases with gastrointestinal tract involvement. There were 29 males (69.0%) and 13 females (31.0%) with 15 of these cases involving the stomach (35,7%) [10–24], 7 the small intestine (16.7%) [7, 25–30], 5 the esophagus (11.9%) [6, 31–34], 5 the colon (11.9%) [35–37], 5 the rectum (11.9%) [38–42], 4 the gallbladder (9.5%) [43–46], and in 1 case a gastrojejunal anastomosis (2.4%) [47].

Of these cases, only 7 succumbed to hemorrhage from the vascular lesion, with 5 having had evidence of gastrointestinal bleeding (either hematemesis, hematochezia, melena, or a combination) [10, 12, 16, 19, 20, 25, 31]. There were 5 males and 2 females in this group with bleeding from the stomach (*N* = 5), the esophagus (*N* = 1) and the duodenum (*N* = 1). The age range was 12–79 yrs (mean 52.3 yrs). The remaining deaths were due to comorbidities, such as cardiovascular disease, chronic kidney disease, diabetes mellitus, and hypertension.

Thus, although Dieulafoy lesion is a rare form of vascular pathology it may on occasion have lethal consequences. The relatively small size of the damaged vessel may contribute to difficulties in identifying the bleeding point at autopsy, particularly if there is a cap of adherent clot. The relative rarity of this entity makes knowledge of the characteristic appearance and features at autopsy important.
